# 基于重复NGS检测的非小细胞肺癌驱动基因状态变化与稳定性分级

**DOI:** 10.3779/j.issn.1009-3419.2026.106.18

**Published:** 2026-07-20

**Authors:** Dan ZHAO, Fudong XU, Lili ZHANG, Nana ZHANG, Zichen LIU, Kun LI, Chen ZHANG, Lijuan ZHOU, Yujie DONG, Jing MU, Nanying CHE

**Affiliations:** 101149 北京，首都医科大学附属北京胸科医院/北京市结核病胸部肿瘤研究所病理科; Department of Pathology, Beijing Chest Hospital, Capital Medical University/Beijing Tuberculosis and Thoracic Tumor Research Institute, Beijing 101149, China

**Keywords:** 肺肿瘤, 驱动基因, 下一代测序, 基因状态稳定性, 纵向研究, Lung neoplasms, Driver genes, Next-generation sequencing, Gene status stability, Longitudinal studies

## Abstract

**背景与目的:**

下一代测序（next-generation sequencing, NGS）驱动基因检测已成为非小细胞肺癌（non-small cell lung cancer, NSCLC）分子分型与精准治疗的常规手段。动态监测有助于早期识别耐药相关分子特征，并为治疗策略调整提供依据。然而，目前重复NGS检测的稳定性尚缺乏统一的评价标准，且福尔马林固定石蜡包埋（formalin-fixed paraffin-embedded, FFPE）标本NGS用于时序监测的适用范围亦不明确。本研究旨在建立NSCLC驱动基因重复检测的状态稳定性分级体系，为临床制定重复活检策略提供循证依据。

**方法:**

回顾性收集2019年6月至2026年4月于首都医科大学附属北京胸科医院接受不少于2次FFPE组织标本NGS检测的1232例NSCLC患者临床与分子检测数据，初检与末检间隔≥4个月以确保时序分析的代表性，最终纳入942例作为主队列。采用Kappa一致性检验评估9种核心驱动基因[表皮生长因子受体（epidermal growth factor receptor, EGFR）、Kirsten大鼠肉瘤病毒癌基因同源物（Kirsten rat sarcoma viral oncogene homolog, KRAS）、间变性淋巴瘤激酶（anaplastic lymphoma kinase, ALK）、c-ROS肉瘤致癌因子-受体酪氨酸激酶（ROS proto-oncogene 1, receptor tyrosine kinase, ROS1）、间质-上皮细胞转化因子（mesenchymal-epithelial transition factor, MET）、重排期间表达基因（rearranged during transfection, RET）、V-raf鼠肉瘤病毒癌基因同源体B1（v-raf murine sarcoma viral oncogene homolog B1, BRAF）、erb-b2受体酪氨酸激酶2（erb-b2 receptor tyrosine kinase 2, ERBB2）、磷脂酰肌醇-4,5-二磷酸3-激酶催化亚基α（phosphatidylinositol-4,5-bisphosphate 3-kinase catalytic subunit alpha, PIK3CA）]的状态稳定性并构建五级分级体系；采用Wilcoxon符号秩检验比较配对样本变异等位基因频率（variant allele frequency, VAF）差异；通过二元Logistic回归模型筛选突变累积的独立影响因素。

**结果:**

9种基因的状态稳定性分为5级：EGFR高度稳定（Kappa=0.838）；ROS1、ALK、KRAS为较好稳定；BRAF、PIK3CA、RET为中度稳定；ERBB2为低度稳定；MET为高度不稳定。MET状态改变率最高（9.3%），原始观察一致率达90.7%，其Kappa值为0.172，受低患病率（3.7%）压缩效应影响，需结合原始一致率（90.7%）与患病率及偏倚校正Kappa（prevalence-adjusted and bias-adjusted Kappa, PABAK）综合解读。PIK3CA的VAF显著升高（P=0.005）。T790M阳性率由5.8%升至10.8%；末次检测新增30例C797S突变，其中13例合并T790M，17例不伴T790M。主队列总体驱动基因变异新增率为18.0%。二元Logistic回归分析显示，初检突变基因数较少是变异新增的独立影响因素[比值比（odds ratio, OR）=0.399，P<0.001]，性别、检测间隔均无独立关联。

**结论:**

本研究建立了NSCLC驱动基因重复检测的状态稳定性五级分级体系。MET状态改变最为多见，解读时需结合患病率效应综合判断；PIK3CA VAF升高为观察性发现，其临床意义有待前瞻性研究验证。初检突变负荷可反映肿瘤克隆复杂度，对后续变异风险具有提示作用。FFPE标本NGS可用于临床治疗节点的重复监测。

## Abstract (en)

**Background and objective:**

Next-generation sequencing (NGS)-based driver gene testing has become a routine component of molecular subtyping and precision therapy for non-small cell lung cancer (NSCLC). Dynamic genomic monitoring facilitates early detection of resistance-related molecular alterations and informs timely therapeutic adjustments. However, standardized criteria for evaluating the stability of serial NGS testing are currently lacking, and the applicability of NGS using formalin-fixed paraffin-embedded (FFPE) specimens for dynamic monitoring remains poorly defined. This study aims to establish a stability grading system for driver gene status alterations based on repeated NGS testing, and to provide evidence-based support for clinical repeat biopsy strategies.

**Methods:**

Data from 1232 patients with NSCLC who underwent two or more NGS tests on FFPE tissue specimens at Beijing Chest Hospital between June 2019 and April 2026 were collected retrospectively. Patients with an interval of ≥4 months between the initial and last tests were included to ensure the representativeness of temporal analysis, resulting in a main analysis cohort of 942 patients. The Kappa consistency test was used to evaluate the state stability of nine core driver genes [epidermal growth factor receptor (EGFR), Kirsten rat sarcoma viral oncogene homolog (KRAS), anaplastic lymphoma kinase (ALK), ROS proto-oncogene 1, receptor tyrosine kinase (ROS1), mesenchymal-epithelial transition factor (MET), rearranged during transfection (RET), v-raf murine sarcoma viral oncogene homolog B1 (BRAF), erb-b2 receptor tyrosine kinase 2 (ERBB2), and phosphatidylinositol-4,5-bisphosphate 3-kinase catalytic subunit alpha (PIK3CA)] and to construct a five-level grading system. Paired variant allele frequency (VAF) differences were compared using the Wilcoxon signed-rank test. Independent influencing factors for mutation accumulation were identified by binary Logistic regression.

**Results:**

The state stability of the nine genes was classified into five levels: EGFR showed high stability (Kappa=0.838), ROS1/ALK/KRAS good stability, BRAF/PIK3CA/RET moderate stability, and ERBB2 low stability, and MET showed high instability. MET exhibited the highest rate of state change (9.3%) with a raw observed agreement of 90.7%. Its Kappa value (0.172) was influenced by the low prevalence (3.7%) compression effect and should therefore be interpreted alongside the observed agreement (90.7%) and the prevalence-adjusted and bias-adjusted Kappa (PABAK). The VAF of PIK3CA increased significantly (P=0.005). T790M positivity increased from 5.8% to 10.8%, and 30 new C797S mutations were detected at the last test (13 with T790M, 17 without). The overall rate of new driver gene variants in the main cohort was 18.0%. Binary Logistic regression showed that a lower number of initial mutated genes was the only independent predictor of new variants [odds ratio (OR)=0.399, P<0.001], while sex and detection interval showed no independent association.

**Conclusion:**

A five level stability grading system for state changes of driver genes in NSCLC based on repeated NGS testing has been established. MET showed the most frequent state changes, which should be interpreted in conjunction with the prevalence effect. The VAF increase of PIK3CA is an observational finding, and its clinical significance requires further prospective validation. A lower initial mutation burden may reflect tumor clonal complexity and was associated with a higher likelihood of subsequent acquisition of new variants. FFPE based NGS is applicable for repeated testing at clinical treatment decision nodes.

肺癌是我国发病率和死亡率最高的恶性肿瘤。基于下一代测序（next-generation sequencing, NGS）的驱动基因检测，现已成为非小细胞肺癌（non-small cell lung cancer, NSCLC）分子分型与精准治疗的常规手段^[1,2]^。动态监测驱动基因状态变化，有助于尽早发现耐药相关分子特征、指导治疗方案调整并评估疾病预后，在NSCLC临床诊疗中具有重要意义^[3]^。

目前动态检测的数据主要以液体活检为主，组织标本再活检存在很多临床实际问题^[4]^，因此基于组织标本驱动基因时序状态变化的数据仍有较多不足，存在3个问题：（1）尚无统一质控标准，无法有效区分同步多灶活检的空间异质性与病灶时序改变，易造成研究结果偏倚^[5]^；（2）针对我国人群高发的9种核心驱动基因，仍缺少大样本、严格质控的纵向配对数据，难以指导临床合理开展重复活检^[6,7]^；（3）甲醛固定石蜡包埋（formalin-fixed paraffin-embedded, FFPE）组织标本NGS用于时序监测的适用范围，仍缺乏充分循证依据 ^[8]^。

本研究基于1232例NGS配对队列（其中主队列942例），通过设置4个月检测间隔的严格质控标准及分层分析，旨在建立NSCLC驱动基因状态稳定性分级体系，明确FFPE标本NGS用于治疗决策节点时序监测的临床价值，从而为临床重复活检策略的制定及后续同类研究提供循证依据。研究所纳入的9种核心驱动基因[表皮生长因子受体（epidermal growth factor receptor, EGFR）、Kirsten大鼠肉瘤病毒癌基因同源物（Kirsten rat sarcoma viral oncogene homolog, KRAS）、间变性淋巴瘤激酶（anaplastic lymphoma kinase, ALK）、c-ROS肉瘤致癌因子-受体酪氨酸激酶（ROS proto-oncogene 1, receptor tyrosine kinase, ROS1）、间质-上皮细胞转化因子（mesenchymal-epithelial transition factor, MET）、重排期间表达基因（rearranged during transfection, RET）、V-raf鼠肉瘤病毒癌基因同源体B1（v-raf murine sarcoma viral oncogene homolog B1, BRAF）、erb-b2受体酪氨酸激酶2（erb-b2 receptor tyrosine kinase 2, ERBB2）、磷脂酰肌醇-4,5-二磷酸3-激酶催化亚基α（phosphatidylinositol-4,5-bisphosphate 3-kinase catalytic subunit alpha, PIK3CA）]均依据我国人群NSCLC FFPE标本NGS大样本突变谱数据确定^[9]^，具体选择标准为：（1）基于我国人群NSCLC大样本突变谱数据的流行病学证据（突变频率≥1%）；（2）靶向治疗价值，已经有国家药品监督管理局（National Medical Products Administration, NMPA）或美国食品药品监督管理局（Food and Drug Administration, FDA）批准或者处于关键临床试验阶段的靶向药物；（3）指南推荐：被美国国家综合癌症网络（National Comprehensive Cancer Network, NCCN）列为常规检测靶点。这9种基因覆盖了我国NSCLC人群约80%的可靶向驱动变异，符合精准医学背景下的临床检测需求。

## 1 资料与方法

### 1.1 研究对象

本研究回顾性纳入2019年6月至2026年4月于首都医科大学附属北京胸科医院行FFPE标本NGS检测的NSCLC患者。纳入标准：（1）病理明确诊断为NSCLC；（2）接受不少于2次FFPE标本NGS检测。排除标准：（1）标本DNA质量不达标；（2）同一病理编号下的同步多灶活检标本（排除空间异质性对时序分析的干扰）；（3）国际肺癌研究协会（International Association for the Study of Lung Cancer, IASLC）提出的肺腺癌原发灶与肺内转移灶的病理及分子鉴别共识中的临床-病理基本原则^[10]^，明确提示为多原发肺癌的病例，如2次检测对应的病灶具有不同组织学类型（如腺癌 vs 鳞状细胞癌），或病灶为微浸润性肺腺癌等。本研究最终共纳入1232例患者，经首都医科大学附属北京胸科医院伦理委员会审批（No.2025-008），回顾性研究豁免知情同意。所有研究流程符合《赫尔辛基宣言》伦理要求。

本研究参考国际公认的4个月时间阈值，以2次检测间隔4个月为界进行分组：检测间隔≥4个月者设为主队列，共942例；间隔<4个月者设为副队列，共290例，主队列基线资料详见表1。主分析基于942例主队列开展，以排除短间隔内可能存在的瘤内异质性或技术误差对时序变化分析的干扰；敏感性分析则纳入全部1232例患者，用于验证研究结论的稳健性。本研究将初检全阴性定义为9种核心驱动基因均未检出点突变、插入缺失、基因融合及拷贝数扩增等各类变异。

**表1:** 942例主队列临床病理特征与9种驱动基因检出率

Clinical characteristics	n	EGFR, n (%)	KRAS, n (%)	ALK, n (%)	ROS1, n (%)	MET, n (%)	RET, n (%)	BRAF, n (%)	ERBB2, n (%)	PIK3CA, n (%)
Sex										
Male	475	239 (50.3)	59 (12.4)	26 (5.5)	2 (0.4)	16 (3.4)	13 (2.7)	17 (3.6)	10 (2.1)	38 (8.0)
Female	467	375 (80.3)	20 (4.3)	30 (6.4)	9 (1.9)	19 (4.1)	5 (1.1)	14 (3.0)	12 (2.6)	43 (9.2)
Age										
<65 yrs	532	347 (65.2)	44 (8.3)	43 (8.1)	8 (1.5)	18 (3.4)	14 (2.6)	17 (3.2)	14 (2.6)	43 (8.1)
≥65 yrs	410	267 (65.1)	35 (8.5)	13 (3.2)	3 (0.7)	17 (4.1)	4 (1.0)	14 (3.4)	8 (2.0)	38 (9.3)
Histological type										
Adenocarcinoma	832	596 (71.6)	77 (9.3)	52 (6.2)	11 (1.3)	32 (3.8)	17 (2.0)	27 (3.2)	20 (2.4)	69 (8.3)
Others	110	18 (16.4)	2 (1.8)	4 (3.6)	0 (0.0)	3 (2.7)	1 (0.9)	4 (3.6)	2 (1.8)	12 (10.9)
Total	942	614 (65.2)	79 (8.4)	56 (5.9)	11 (1.2)	35 (3.7)	18 (1.9)	31 (3.3)	22 (2.3)	81 (8.6)

EGFR: epidermal growth factor receptor; KRAS: Kirsten rat sarcoma viral oncogene homolog; ALK: anaplastic lymphoma kinase; ROS1: ROS proto-oncogene 1, receptor tyrosine kinase; MET: mesenchymal-epithelial transition factor; RET: rearranged during transfection; BRAF: v-raf murine sarcoma viral oncogene homolog B1; ERBB2: erb-b2 receptor tyrosine kinase 2; PIK3CA: phosphatidylinositol-4,5-bisphosphate 3-kinase catalytic subunit alpha.

### 1.2 标本处理与检测方法

本研究全部采用FFPE标本，所有标本均经2名高年资病理医师双盲阅片，确认肿瘤细胞含量≥20%后，进行切片与核酸提取。

#### 1.2.1 DNA与RNA提取

采用核酸提取或纯化试剂（中国天津诺禾致源生物信息科技有限公司）从FFPE的组织切片中提取DNA和RNA。使用Qubit 3.0荧光测定仪（美国Life Technologies公司）对提取所得DNA和RNA的浓度进行定量分析。

#### 1.2.2 NGS文库构建与测序

本研究同时构建DNA和RNA 文库：RNA文库用于检测基因融合（ALK、ROS1、RET），DNA文库用于检测点突变、插入缺失和拷贝数扩增。DNA和RNA文库采用人EGFR、KRAS、BRAF、PIK3CA、ALK、ROS1基因突变检测试剂盒（半导体测序法）（中国天津诺禾致源生物信息科技有限公司）按制造商说明书构建，该检测panel可覆盖60个肺癌相关基因的重要外显子区和内含子区。本研究重点分析中国人群高发的9种核心驱动基因（EGFR、KRAS、ALK、ROS1、MET、RET、BRAF、ERBB2、PIK3CA）。各基因检出的变异类型包括：EGFR（点突变、插入缺失）、KRAS（点突变、插入缺失）、ALK（融合、点突变）、ROS1（融合、点突变、插入缺失）、MET（点突变、插入缺失、外显子14跳跃突变、拷贝数扩增）、RET（融合、点突变、插入缺失）、BRAF（点突变、插入缺失）、ERBB2（点突变、插入缺失、拷贝数扩增）、PIK3CA（点突变）。使用Qubit 3.0荧光计（美国Life Technologies公司）和ABI 7500实时荧光定量聚合酶链式反应（polymerase chain reaction, PCR）系统（美国Thermo Fisher Scientific公司）对构建的文库进行定量。测序工作在DA8600高通量测序仪（中国广州达安基因股份有限公司）完成。研究期间测序平台、检测试剂盒及生信分析流程均未发生变更，以确保数据的可比性。

#### 1.2.3 测序质控指标

DNA文库平均测序深度>2000×；RNA文库序列数>60,000条。本研究采用扩增子法建库，该方法检测拷贝数变异（copy number variation, CNV）流程简便，但对低水平扩增（拷贝系数CNV<2倍）的灵敏度略低于捕获法，因此本研究仅纳入CNV≥2倍的扩增作为阳性。需要指出的是，本研究未根据肿瘤细胞纯度计算癌细胞分数（cancer cell fraction, CCF），VAF的动态变化可能受到2次活检样本间肿瘤细胞纯度差异的影响，因此VAF结果仅供探索性参考。

#### 1.2.4 生物信息学分析

原始测序数据经SAMtools转换格式、排序及索引后，使用TMAP比对工具与人类参考基因组（GRCh37/hg19）进行比对。采用Torrent Variant Caller（TVC）和MuSCAN两种工具互补检测单核苷酸变异（single nucleotide variants, SNVs）及小片段插入缺失（insertions and deletions, InDels），经ANNOVAR和SnpEff 进行功能注释后，筛选具有临床意义的突变。

### 1.3 统计学方法

采用SPSS 26.0及R 4.2.0软件进行数据分析。计数资料以频数（百分比）描述，组间比较采用χ^2^检验或Fisher精确检验；计量资料以中位数、范围或四分位数间距（interquartile range, IQR）描述，配对样本比较采用 Wilcoxon符号秩检验，并报告标准化Z值。采用Kappa检验评价2次检测结果的一致性，计算Kappa值、渐近标准误、Z值、P值及95%CI。一致性分级标准：Kappa>0.8为高度稳定，0.6-0.8为较好稳定，0.4-0.6为中度稳定，0.2-0.4为低度稳定，<0.2为高度不稳定。鉴于Kappa值易受基因患病率影响，低阳性率基因可能出现“原始一致率高但Kappa值偏低”的现象，本研究同步报告原始观察一致率（Po）作为辅助判断指标，并计算患病率及偏倚校正Kappa（prevalence-adjusted and bias-adjusted kappa, PABAK）以校正患病率效应。

本研究稳定性分级以原始Kappa值作为唯一判定标准，PABAK和原始一致率（Po）仅作为辅助参考指标，用于校正低患病率基因的统计学偏差。多因素分析采用二元Logistic回归模型。因变量定义为末检较初检新增至少1种驱动基因变异（是=1，否=0），具体包括两类情况：（1）初检阴性、末检转为阳性；（2）初检检出点突变/插入缺失/融合，末检新增拷贝数扩增（CNV≥2倍）。将单因素分析中P<0.05的变量纳入多因素模型，计算比值比（odds ratio, OR）及95%CI，采用Hosmer-Lemeshow检验评价模型拟合效果。采用Cochran-Armitage趋势检验分析检测间隔与突变累积的剂量反应关系，并将检测间隔作为连续变量纳入二元Logistic回归。所有统计检验均为双侧检验，检验水准α=0.05。P<0.05为差异具有统计学意义。

## 2 结果

### 2.1 队列基线特征

主队列942例患者中，男性475例（50.4%），女性467例（49.6%）；年龄中位数为63岁（范围：29-86岁），<65岁532例（56.5%），≥65岁410例（43.5%）。检测次数：2次746例（79.2%），3次158例（16.8%），≥4次38例（4.0%）。初检与末检间隔中位数为16.9个月（范围：4.0-78.1个月），4-6个月79例（8.4%），6-12个月238例（25.3%），12-24个月306例（32.5%），>24个月319例（33.9%）。组织学类型：腺癌832例（88.3%）、鳞状细胞癌95例（10.1%）、腺鳞癌9例（1.0%）、肉瘤样癌6例（0.6%）。初检驱动变异阳性804例（85.4%），EGFR阳性614例（65.2%）。各驱动基因检出率在不同临床亚组间呈现显著差异（表1）：EGFR突变在女性（80.3%）和腺癌（71.6%）患者中检出率显著高于男性（50.3%）和非腺癌（16.4%）患者；KRAS突变则呈现相反趋势（男性12.4% vs 女性4.3%）；ALK融合在<65岁患者中检出率（8.1%）约为≥65岁（3.2%）的2.5倍。上述分布特征与我国人群NSCLC驱动基因流行病学数据一致。

### 2.2 驱动基因纵向稳定性分级

基于配对检测的状态改变率与Kappa一致性，本研究将9种核心驱动基因分为5个稳定性等级（表2）。EGFR为高度稳定基因（Kappa: 0.838, 95%CI: 0.804-0.872），单次初检结果可靠性高；ROS1、ALK和KRAS为较好稳定基因（Kappa: 0.6-0.8）；BRAF、PIK3CA 和RET为中度稳定基因（Kappa: 0.4-0.6）；ERBB2为低度稳定基因（Kappa: 0.2-0.4）；MET为高度不稳定基因（Kappa: 0.172）。上述结果提示，临床可根据基因稳定性差异制定个体化的重复检测策略。

**表2:** 9种核心驱动基因状态变化稳定性分级（n=942）

Gene	Initial positive rate (%)	Final positive rate (%)	State change rate (%)	Kappa	95%CI	Z	P	Observed agreement (%)	PABAK	Stability grading
EGFR	65.2	63.9	7.4	0.838	0.804-0.872	49.85	<0.0001	92.6	0.851	High stability
ROS1	1.2	1.6	0.8	0.688	0.479-0.897	6.46	<0.0001	99.2	0.984	Good stability
ALK	5.9	7.0	3.2	0.737	0.645-0.829	15.63	<0.0001	96.8	0.936	Good stability
KRAS	8.4	8.2	4.7	0.693	0.606-0.779	15.52	<0.0001	95.3	0.906	Good stability
BRAF	3.3	3.8	3.9	0.428	0.237-0.618	4.40	<0.0001	96.1	0.921	Moderate stability
PIK3CA	8.6	8.9	8.0	0.502	0.395-0.609	9.17	<0.0001	92.0	0.840	Moderate stability
RET	1.9	2.4	2.4	0.427	0.189-0.664	3.51	0.0004	97.6	0.952	Moderate stability
ERBB2	2.3	3.8	4.0	0.325	0.108-0.542	2.92	0.0040	96.0	0.919	Low stability
MET	3.7	8.2	9.3	0.172	0.014-0.330	2.14	0.0330	90.7	0.813	High instability

Grading is based on Kappa values: Kappa>0.8 indicates high stability; 0.6-0.8 indicates good stability; 0.4-0.6 indicates moderate stability; 0.2-0.4 indicates low stability; and Kappa<0.2 indicates high instability. PABAK and observed agreement (Po) are auxiliary reference indicators used to correct for the prevalence effect. Po=(number of consistently negative cases+number of consistently positive cases)/total number of cases. PABAK: prevalence-adjusted and bias-adjusted Kappa; CI: confidence interval.

MET显示最高的状态改变率（9.3%, 88/942），其Kappa值为0.172（95%CI: 0.014-0.330, Z=2.14, P=0.033），处于高度不稳定区间。需特别说明的是，MET的原始观察一致率（Po）高达90.7%（854/942），其Kappa值偏低主要受低患病率（初检阳性率仅3.7%，35/942）的固有压缩效应影响；经患病率校正后PABAK为0.813，提示MET检测本身的重复性良好，其“高度不稳定”的统计学判定反映的是低基线阳性背景下新发扩增/突变的临床现象，而非检测技术的不稳定性。初检MET阴性者末检阴性转阳率达7.2%（65/907），阴性转阳病例占所有MET状态改变病例的73.9%（65/88）。在65例阴性转阳患者中，37例为拷贝数扩增（CNV≥2倍），22例为点突变，5例为插入缺失，1例为MET外显子14跳跃突变，提示状态改变主要来自初检阴性患者末检时新检出的多种变异类型，临床在疾病进展时应重点关注MET状态复查^[11]^。

### 2.3 VAF动态变化特征

VAF配对比较（表3）显示，PIK3CA突变丰度显著升高（初检中位数15.58% vs 末检23.62%，ΔVAF=+8.04%，95%CI: +2.51%-+13.52%，Z=2.81，P=0.005），PIK3CA VAF动态升高为观察性发现，鉴于配对样本量较小（n=45）且未校正肿瘤纯度和治疗背景，不宜直接推断其为耐药机制或早期耐药监测指标；该现象可能与治疗压力下的亚克隆扩增有关，但需在明确治疗背景的前瞻性研究中验证。EGFR VAF呈下降趋势（28.80% vs 25.67%, Δ=-3.13%，95%CI: -7.10%-+0.84%, P=0.124, n=573），初检阳性614例中41例因仅检出基因扩增（无VAF值）或数据缺失未纳入配对。虽无统计学差异，但结合状态转移分析（阳性转阴41例，阴性转阳29例），提示敏感克隆在治疗压力下被部分清除，而耐药克隆可能通过亚克隆扩增代偿性维持。KRAS（ΔVAF=+2.56%, P=0.071）、ALK（ΔVAF=-1.16%, P=0.910）及ERBB2（ΔVAF=-4.29%, P=0.846）的VAF变化无统计学差异，考虑与样本量有限导致的检验效能不足有关。

**表3:** 驱动基因VAF动态变化（Wilcoxon符号秩检验）

Gene	n	Median VAF at initial testing (IQR), %	Median VAF at final testing (IQR), %	ΔVAF, %	95%CI, %	Z	P
EGFR^#^	573	28.80 (16.12-47.28)	25.67 (13.71-45.83)	-3.13	-7.10-+0.84	-1.53	0.124
KRAS	56	20.48 (10.15-32.49)	23.04 (12.38-40.65)	+2.56	-2.54-+7.78	1.80	0.071
ALK	12	42.17 (38.92-50.64)	41.01 (38.41-46.84)	-1.16	-6.35-+4.05	-0.11	0.910
BRAF^†^	15	-	-	-	-	-	-
ERBB2	10	24.77 (15.58-42.68)	20.48 (12.95-38.50)	-4.29	-12.49-+3.89	-0.19	0.846
PIK3CA*	45	15.58 (7.53-27.34)	23.62 (11.56-33.47)	+8.04	+2.51-+13.52	2.81	0.005

*81 patients were initially positive for PIK3CA, among whom 45 had paired VAF data at both initial and final testing. The remaining 36 were excluded from paired analysis due to conversion from positive to negative at final testing (no final VAF value available). ^#^Paired VAF analysis for EGFR included 573 patients. The remaining 41 were excluded due to detection of only gene amplification (no VAF value) or missing data. ^†^BRAF was not included in the analysis due to insufficient paired VAF data. VAF: variant allele frequency; IQR: interquartile range.

### 2.4 初检全阴性患者的再检出特征

初检全阴性138例（14.6%），复检检出至少1种驱动突变44例（31.9%），复检仍阴性94例（68.1%）。复检阳性中ALK 18例（40.9%）居首，其次为RET 8例（18.2%）、ROS1 17例（15.9%）、BRAF 5例（11.4%）、EGFR 4例（9.1%）、PIK3CA 4例（9.1%）、ERBB2 2例（4.5%）、KRAS 2例（4.5%）、MET 3例（6.8%）。突变基因数分布：1种34例（77.3%），2种7例（15.9%），≥3种3例（6.8%）。该结果提示，即使初检全阴性，疾病进展时重复活检仍有31.9%的概率检出可靶向驱动突变。

### 2.5 耐药突变时序累积特征

T790M耐药突变初检阳性55例（5.8%），末检阳性102例（10.8%），净增加47例（增幅 85.5%）；其中持续阳性28例，阳性转阴27例（提示耐药克隆清除或异质性丢失），阴性转阳74例（提示耐药克隆时序累积）。初检T790M阳性的55例均同时携带其他EGFR突变，其中54例为经典敏感突变19del或L858R，1例为非经典突变V769dup合并T790M；8例（14.5%）同时携带C797S突变。

C797S初检阳性8例（0.8%），末检阳性38例（4.0%），净增加30例（增幅375.0%）；其中持续阳性8例，无阳性转阴病例，阴性转阳30例。末检新增的30例C797S中，13例伴随T790M阳性，17例未检出T790M。13例伴T790M者中，顺式10例，反式3例；进一步分析显示，其中5例初检已携带T790M（序贯耐药模式），8例与C797S同步于末检首次出现（同步获得模式，即de novo），提示C797S的获得可能既包括T790M驱动下的序贯耐药，也包括治疗压力下的de novo同步出现。17例无T790M者提示C797S的出现可能不依赖T790M，但受治疗数据缺失限制无法进一步分析^[12]^。

### 2.6 突变基因数增加的影响因素

单因素分析（表4）显示，初检突变基因数与驱动基因变异新增显著关联（OR=0.399, 95%CI: 0.289-0.551, P<0.001），每增加1个初检突变基因，后续新增变异的风险降低约60%。性别（男性: OR=1.164, 95%CI: 0.834-1.623, P=0.371）、年龄≥65岁（OR=1.124, 95%CI: 0.805-1.569, P=0.493）、检测间隔12-24个月（OR=1.322, 95%CI: 0.936-1.868, P=0.113）及检测间隔>24个月（OR=0.982, 95%CI: 0.691-1.396, P=0.919）均未显示统计学差异。多因素分析（表4）纳入单因素分析中P<0.05的变量（仅初检突变基因数），结果显示初检突变基因数（OR=0.399, 95%CI: 0.289-0.551, P<0.001）是突变基因数增加的独立影响因素。即初检突变基因数较少的患者更易出现后续驱动基因变异增加，提示初检突变负荷可能反映肿瘤克隆复杂度——突变负荷低者可能以单一驱动克隆为主，后续更易因治疗选择压力或自然演变而获得新的驱动变异；而突变负荷高者可能已具有多克隆并存特征，后续新增可检出的驱动变异空间相对有限。

**表4:** 突变基因数增加的Logistic回归分析

Variable	Univariate analysis				Multivariate analysis		
	OR	95%CI	P		OR	95%CI	P
Male (vs Female)	1.164	0.834-1.623	0.371		-	-	-
Age ≥65 yrs (vs <65 yrs)	1.124	0.805-1.569	0.493		-	-	-
Test interval 12-24 months (vs <12 months)	1.322	0.936-1.868	0.113		-	-	-
Test interval >24 months (vs <12 months)	0.982	0.691-1.396	0.919		-	-	-
Number of mutations at initial testing (per 1 increase)	0.399	0.289-0.551	<0.001		0.399	0.289-0.551	<0.001

The reference group for test interval was <12 months (combining the 4-6 months and 6-12 months subgroups). OR: odds ratio. Only variables with P<0.05 in univariate analysis were included in the multivariate model.

### 2.7 敏感性分析

纳入全部1232例进行敏感性分析（表5），副队列（间隔<4个月，n=290）与主队列（间隔≥4个月，n=942）比较显示：全队列突变基因数增加率为17.8%（219/1232），子集阳性例数不超过全集（n=170 vs n=220），数据逻辑一致；<4个月亚组（n=290）的突变增加率为16.9%（49/290），MET变化率也降至2.5%，均显著低于主队列。该结果提示副队列中同步多灶分期活检或同阶段重复检测稀释了时序状态变化信号，掩盖了真实的MET不稳定性，进一步证实≥4个月间隔标准对于排除同步多灶混杂的必要性。

**表5:** 敏感性分析对比（主分析 vs 全队列 vs 副队列）

Indicator	Main analysis cohort ( n=942)	Full cohort (n=1232)	Secondary cohort (n=290)
Rate of new driver gene variants (%)	18.0 (170/942)	17.8 (219/1232)	16.9 (49/290)
Rate in males (%)	19.2 (91/475)	18.9 (117/619)	18.1 (26/144)
Rate in females (%)	16.9 (79/467)	16.6 (102/613)	15.8 (23/146)
MET state change rate (%)	9.3	7.6	2.5
Sex difference	Not significant	Not significant	Not significant

## 3 讨论

### 3.1 核心学术贡献

本研究基于国内大样本单中心FFPE配对队列，明确4个月检测间隔作为质控基准，建立了NSCLC驱动基因重复NGS检测的状态变化分析框架，并构建中国人群9种核心驱动基因的状态变化分级体系，同时评估了FFPE标本NGS检测在临床治疗决策节点重复检测的应用价值。既往国内多项重复NGS相关研究未对同步多灶活检与时序样本设置明确区分标准，易造成数据分析偏倚。本研究参考国际公认的4个月时间阈值划分检测间隔，敏感性分析结果显示：检测间隔<4个月亚组中MET状态改变率明显下降，证实短间隔样本易受同步多灶空间异质性干扰。本研究确立的质控方案，可为国内同类检测分析研究提供方法学参考。

### 3.2 稳定性分级的临床价值

本研究在严格质控的前提下，完成9种核心驱动基因重复检测的状态变化数据分析。其中EGFR一致性最佳，单次检测结果可靠性高；MET的Kappa值最低。统计结果显示，初检MET阴性人群中，阴性转阳占MET所有状态改变病例的73.9%（65/88），提示MET状态改变主要以新发突变或扩增为主，临床随访进展时需重点关注该基因复检。

需说明的是，Kappa指标存在患病率效应：基因阳性率偏低时，即便原始一致率较高，Kappa值也会被低估。本研究中MET初检阳性率仅3.7%，原始观察一致率达90.7%，经患病率与偏倚校正后PABAK=0.813，提示MET本身检测重复性良好，Kappa偏低是统计学效应所致，并非检测技术缺陷。MET的状态改变，主要体现为疾病进程中新发扩增或突变的生物学现象。针对MET扩增类结果，由于FFPE标本DNA易出现碎片化问题，CNV检测存在批次差异，建议后续结合荧光原位杂交（fluorescence in situ hybridization, FISH）开展交叉验证，区分真实扩增与标本质量、检测体系带来的假阳性^[13,14]^。初检MET阴性者（n=907）中，末检转阳65例，阴性转阳率为7.2%（65/907）。这65例阴性转阳病例占MET全部状态改变病例的73.9%（65/88），提示MET状态改变主要来自初检阴性患者在随访中新增的突变或扩增。65例转阳者的变异亚型分布为：拷贝数扩增37例，点突变或插入缺失22例，插入缺失5例，外显子14跳跃突变1例，以拷贝数扩增和点突变/插入缺失为主要类型。临床在疾病进展时应对初检MET阴性、末检转阳的患者重点复查MET状态。

本研究观察到PIK3CA的VAF水平显著升高（P=0.005），该现象既往被认为可能与靶向治疗作用下的亚克隆扩增有关，但其因果关系仍缺乏直接证据；同时，PIK3CA突变位点分布广泛，本研究未开展位点分层分析，结合本研究缺少完整治疗记录的现状，该指标作为早期监测标志物的价值，仍有待大样本、有明确治疗背景的前瞻性研究进一步验证。

### 3.3 耐药突变时序累积

T790M突变阳性率由5.8%升至10.8%，提示该耐药相关突变随随访时间呈现累积趋势。27例初检T790M阳性患者末次检测转阴，该现象可由治疗干预后克隆清除、采样异质性等多种因素解释；由于本研究未收集用药信息，无法进一步区分具体成因^[15]^。

末次检测新增30例C797S突变，其中13例合并T790M突变，17例未检出T790M突变。已知顺式、反式C797S对不同EGFR-酪氨酸激酶抑制剂（tyrosine kinase inhibitors, TKIs）存在疗效差异，本队列C797S的分布特征与既往第三代EGFR-TKIs耐药人群的分子表型相近，但受治疗数据缺失限制，仅作为观察性描述，不做因果推断^[16]^。

本研究还发现1例特殊病例，该患者携带EGFR外显子20插入突变（V769dup）合并T790M，该类突变对第一代EGFR-TKIs天然耐药^[17]^；随访中该病例在保留T790M的同时新增EGFR 19del突变，为非经典EGFR突变人群的分子状态变化提供了个案参考。

### 3.4 突变累积影响因素的解读

二元Logistic回归结果显示，初检突变基因数量是驱动基因新增变异的独立相关因素（OR=0.399, P<0.001）。从分子特征角度分析，初检突变负荷偏低，往往提示肿瘤以单一克隆为主，在疾病进展过程中更易出现新的基因变异；而高突变负荷肿瘤多为多克隆共存，后续新增可检出驱动变异的空间相对有限。该结果与肿瘤分子演变的相关理论基本相符。

本研究中性别、检测间隔未表现出独立相关性，该现象可能与严格的入组质控标准有关。分析原因如下：（1）本研究设置严格入组与质控标准，剔除同步多灶、标本不合格等混杂样本，减少数据偏倚；（2）检测间隔并非独立影响因素，其背后往往伴随治疗线数、用药方案等混杂变量，在无系统治疗记录的情况下，单纯时间维度的相关性会被掩盖；（3）既往部分队列未对病理类型进行标准化，女性人群腺癌占比、靶向治疗使用率更高，易造成性别指标出现统计学差异，本队列病理构成相对均衡，该混杂效应被削弱。严格的质控与筛选能够过滤干扰信号，得到更贴近真实的基因状态变化数据，避免统计学假象带来的误判。

### 3.5 FFPE标本NGS检测适用边界

FFPE标本NGS检测可用于治疗前基线评估、疾病进展阶段的临床决策节点检测，与循环肿瘤DNA（circulating tumor DNA, ctDNA）液体活检形成互补。本队列中，初检全阴性患者进展后重复活检，仍有31.9%可检出可靶向驱动基因，提示即便基线无驱动变异，疾病进展时仍有必要再次行分子检测。

两种检测手段各有优劣：FFPE标本NGS检测可明确组织病理类型，对基因融合、CNV检测灵敏度更高，但受采样位点限制，无法反映全身肿瘤负荷；ctDNA可体现全身肿瘤分子特征，但对低频突变、CNV检出能力有限。组织与液体活检结果存在10%-30%的不一致性，受条件限制本研究未设置液体活检对照，二者联合应用的最优模式仍需进一步探索。此外，FFPE标本DNA碎片化问题突出，CNV检测假阳性率可达10%-89%，MET、ERBB2等扩增类指标易受标本批次影响，临床解读及后续研究均建议联合FISH验证。

### 3.6 研究的局限性

（1）本研究为回顾性分析，未系统收集两次检测间期的治疗方案、用药种类、治疗线数等数据，无法区分基因状态变化是疾病自然进展还是药物干预所致。PIK3CA丰度改变、T790M累积、MET状态波动等结果，均仅为真实世界观察性结论。Logistic回归模型中检测间隔无统计学意义，也侧面提示治疗是重要混杂因素。（2）研究为单中心回顾性队列，虽设置严格的纳入和排除标准，但仍存在选择偏倚；未对全部排除病例开展基线对比，队列外推性存在一定局限。（3）缺少无进展生存期、总生存期等临床预后终点数据，分级体系对远期预后的预测价值未能验证。（4）本检测平台共覆盖60个肺癌相关基因，本研究仅聚焦9种核心驱动基因；TP53、神经营养性酪氨酸受体激酶（neurotrophic tyrosine receptor kinase, NTRK）、神经母细胞瘤RAS病毒癌基因同源物（neuroblastoma RAS viral oncogene homolog, NRAS）、成纤维细胞生长因子受体（fibroblast growth factor receptor, FGFR）、丝裂原活化蛋白激酶激酶1（mitogen-activated protein kinase kinase 1, MAP2K1）等基因未纳入分析。其中TP53为高频伴随突变，与基因组不稳定性有关，该部分数据缺失会影响对肿瘤整体分子状态变化的全面判断。同时 Panel对部分剪接位点区域覆盖不足，可能存在少量MET外显子14跳跃突变的漏检。（5）未设置液体活检平行对照，无法对比两种检测方式的一致性与互补性。（6）FFPE标本固有缺陷导致CNV检测存在假阳性风险，MET、ERBB2扩增结果需警惕批次效应。（7）研究周期近7年，虽检测平台、试剂盒主体未变更，但探针设计、生信分析流程存在迭代可能，未开展批次效应校正。（8）多因素回归模型未纳入病理类型、临床分期等潜在混杂变量，可能对部分指标效应造成干扰。

本研究基于1232例NSCLC FFPE标本NGS检测配对队列（主队列942例），建立了驱动基因重复检测的状态变化五级分级体系。MET在重复检测中状态改变率最高，解读时需结合原始一致率、PABAK及患病率综合判断；PIK3CA的VAF升高为观察性结果，其临床意义仍有待前瞻性研究验证。初检突变基因数量越少，后续越易出现新增驱动变异，提示基线突变负荷可作为肿瘤分子演变的参考指标。T790M、C797S等耐药突变的累积特征，与既往第三代EGFR-TKIs耐药人群的分子表型相近，但受治疗数据限制仅作描述。性别、检测间隔未成为独立影响因素，考虑与本研究严格质控、混杂因素较少有关。FFPE标本NGS检测适用于NSCLC治疗节点的重复检测，可为临床制定重复活检策略提供观察性循证依据。
